# Molecular adsorbent recirculating system treatment in 69 patients listed for liver transplantation

**DOI:** 10.1186/cc14462

**Published:** 2015-03-16

**Authors:** P Olin, H Haugaa

**Affiliations:** 1 Oslo University Hospital, Oslo, Norway

## Introduction

The molecular adsorbent recirculating system (MARS) is used to remove circulating albumin-bound toxins in patients with liver failure. However, the application of MARS has not demonstrated improved survival in randomized clinical trials and the clinical utility has not been finally established. In our department, the use of MARS is now restricted to the most critically ill patients with acute or acute on chronic liver failure. We aimed to explore MARS efficacy in removing toxicity parameters and the safety of the system.

## Methods

Since 2005, we have treated 69 patients (30 males/39 females with median age of 39 years ranging from 1 month to 70 years) listed for liver transplantation with MARS. The median Model of End Stage Liver Disease (MELD) score in patients older than 12 years of age (*n *= 56) was 33 (interquartile range 26 to 39). The flow rate was 35 to 40 ml/ kg/hour and treatment kits were changed every 8 to 12 hours. The patients were treated for a median of 31 hours (range 1 to 240 hours).

## Results

Fifty-five patients (79%) were successfully bridged to transplantation. Nine died before they could be transplanted, and five patients recovered without liver transplantation. Forty-four (81%) of the transplanted patients were alive 30 days after transplantation. Ammonium decreased modestly from a median of 148 to 124 μM (*P *= 0.03) during MARS treatment. We detected worsening of coagulopathy with significant decreases in platelet count and fibrinogen concentrations, and increase in International Normalized Ratio. Phosphate and magnesium decreased significantly during MARS treatment.

## Conclusion

Close observation and treatment of coagulopathy and electrolyte disturbances is essential when treating patients with MARS. MARS can reduce and stabilize ammonium and other biomedical markers in patients listed for urgent liver transplantation with high MELD score and liver encephalopathy. It seems that, in some cases and with our settings, the detoxification properties of MARS may be insufficient.

